# A Comparative Study of the Management of Stage 2 hypertension by Combined therapy with Losartan, Amlodipine and Hydrochlorothiazide

**Published:** 2012-09-15

**Authors:** Reza Jafarzadeh Esfehani, Azadeh Mahmoodi Gharai, Ali Jafarzadeh Esfehani, Afsaneh Rezaie Kalat, Faezeh Abbasi, Majid Jalalyazdi

**Affiliations:** 1 Education Development Center, Sabzevar University of Medical Sciences, Sabzevar, IR Iran.; 2 Mashhad University of medical science, Mashhad, IR Iran.; 3 Cardiology Department, Sabzevar University of Medical Sciences, Sabzevar, IR Iran.

**Keywords:** Angiotensin Receptor Blocker, Calcium Channel Blocker, Hypertension

## Abstract

**Background:**

The most effective and accurate treatment of hypertensive patients reduces cardiovascular events and improves the quality of life.

**Objective:**

This study compared the efficacy and safety of combined (combination therapy) with an angiotensin-receptor blocker (ARB) a calcium-channel blocker (CCB) (Losartan / Amloidipine 50/10mg) vs maximal combination doses of ARB with hydrochlorothiazide (Losartan /HCTZ 100/25 mg) and maximal combination doses of CCB with HCTZ (Amlodipine /HCTZ 10/25 mg) in the management of stage 2 hypertension.

**Methods:**

This randomized clinical trial (RTC) comprised 478 hypertensive patients with mean age 50.5±5.21 years, and took place between January 2010 and December 2011 in Vasei Hospital clinic in Sabzevar. Antihypertensive drugs were washed out after 5 days of discontinuation of drugs and the patients with mean blood pressure in sitting position ≥ 160 and <200 mmHg in systole and ≥ 100 and <110 mmHg in diastole were randomized into three groups: Losartan / Amlodipine 50/10 mg (n =164) , Losartan / HCTZ 100/25 mg (n =155) and Amlodipine / HCTZ 10/25 mg (n =159). The end point was reaching the blood pressure below 140/90 within 56 days of treatment in each group.

**Results:**

There was a significant difference in systolic blood pressure reductions between treatment groups (P<0.001) and also there was a significant difference between groups in reducing diastolic blood pressure (P<0.01). The highest systolic and diastolic blood pressure reduction respectively was found in Amlodipine/losartane and losartane/HTCZ group. The ANCOVA analysis revealed that only treatment regimen had a significant effect (P=0.01) and other factor including Age, Gender, Diabetes Mellitus, Smoking and High serum cholesterol didn’t have significant effect on blood pressure reduction.

**Conclusion:**

ARB/CCB combination therapy reduced blood pressure more effectively than the maximal doses of ARB or CCB with HCTZ in stage 2 hypertensive patients within this period of study.

## Introduction

Hypertension is one of the most common worldwide diseases afflicting humans and requires meticulous control to produce the maximum reduction in clinical cardiovascular end points. Approximately 26.4% of the adult population worldwide had hypertension in 2000, and this is expected to increase to 29.2% by 2025 (1). The prevalence of hypertension in IR Iran according to the latest meta-analysis study done in 1999 was 21.9 % (2). Hypertension treatment as a disease is an important public health challenge. Achieving recommended goal of blood pressure (BP) (BP <140/90 mmHg or BP <130/80 mmHg in patients with diabetes or chronic kidney disease (3)) is difficult in majority of patients with hypertension 4. There are many drugs available for treatment of hypertension. The variation of Antihypertensive drugs and also extensive clinical studies has led medical practitioners to different ideas for administration of these drugs. The ideal goal is that drug must be efficacious, free from side-effects, enable us to prevent all the complications of hypertension, easy to use and affordable. New onset patients who are diagnosed with uncomplicated hypertension and no compelling indications, choice of first line monotherapy includes angiotensin converting enzyme (ACEI), angiotensin receptor blocker (ARB), calcium channel blocker (CCB) and diuretics (5-7). In patients presenting with stage 2 hypertension or beyond, combination therapy is recommended (8). In this study we evaluated the efficacy rate of combination therapy with ARB and CCB, versus high dose ARB or CCB combined with hydrochlorothiazide (HCTZ) on the patients older than 40 years in stage 2 hypertension.

## Materials and Methods

**Figure1.**
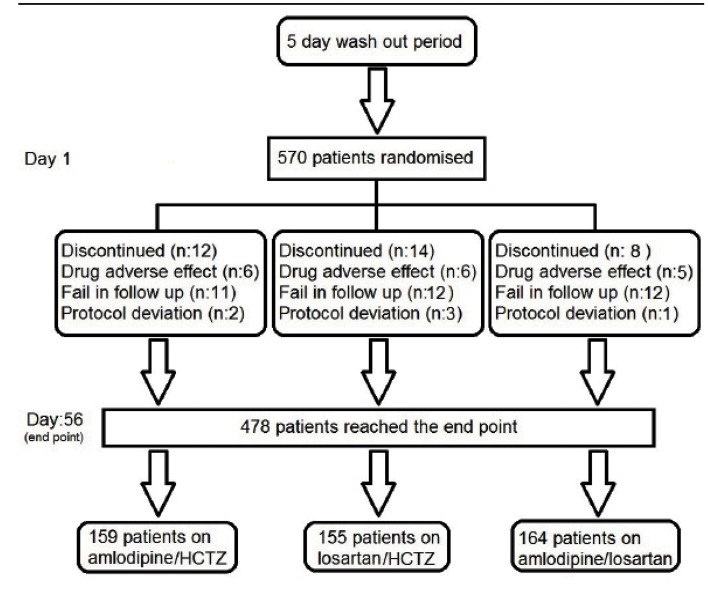
Study design (RCT), concluding 5 days for wash out period and 56 days controlled follow up period. Wash out period was considered in case of incomplete/inadequate treatment, in terms of dosage and type of the drug prior to the intervention. To overcome this confounding issue, we designed a protocol that prescribes routine antihypertensive drugs with proper dosage, based on individual indications, by the cardiologist for 5 days. The prescribed drugs were the first line treatment for hypertension based on patients’ past medical history, drug history and blood pressure at the time of visit. These antihypertensive drugs were Captopril, Enalapril, hydrochlorothiazide, and Metoprolol Here we insist that this primary treatment was performed to ensure that all participants were not under the influence of previous treatments and that the patients were given standard dose of treatment before entering the main study.

This study was a randomized clinical trial on treatment of stage 2 systolic hypertension conducted at Vasei hospital clinic of Sabzevar city in IR Iran between January 2010 and December 2011. The diagnosis and staging of arterial hypertension was based on the mean value of two consecutive office BP measurements that were taken between 17:00 and 20:00 (According to the criteria of JNC 7, before randomization). The study design is summarized in Figure 1. Office measurements of systolic and diastolic BP were performed manually with a calibrated sphygmotensiophone Riester® Sphygmomanometer. In each visit the blood pressure was taken twice, with a 5 minutes gap while the patient is sitting on the chair. The blood pressure was measured in both arms and the higher one is recorded. The patients avoided smoking or taking caffeine at least 30 min before blood pressure measurement. This study enrolled outpatient males and females above 40 years-old who had stage 2 systolic hypertension, deﬁned as a mean in-office sitting systolic BP (MSSBP) of 160 mmHg or greater and less than 200 mmHg or mean sitting diastolic BP (MSDBP) of 100 mmHg and less than 110 mmHg at randomization. Patients were excluded if on mentioned drugs, pregnant or refused to undergo follow-up visits. Treatment was changed to other drugs within one week, if patients exhibited any drug-related adverse effect. Other key exclusion criteria were MSSBP above 200 mmHg or mean sitting diastolic BP (MSDBP) above 110 mmHg at the time of enrollment, use of 4 antihypertensive drugs in the past 30 days, secondary hypertension, or resistant hypertension. Antihypertensive medication were washed out 5 days after discontinuation of drugs and eligible patients were randomized to receive Amlopres (Amlodipine manufactured by Alborz Darou) with maximal dose (5 mg bid) and low dose of Hydrochlorothiazide (25 mg daily) (Amlodipine/HCTZ group), Losartan maximal dose (50 mg bid) and low dose of Hydrochlorothiazide (Losartan /HCTZ group) or Amlodipine 5 mg bid and Losartan 25 mg bid, (Amlodipine Losartan group) (Figure 1). The aim of the study was to compare the change from baseline in MSSBP with the drug regimens at 8 weeks. At each visit, patients were expelled from the study if they had MSSBP above 200 mmHg or MSDBP above 110 mmHg or hypotension (MSSBP <100 mmHg or MSDBP <60 mmHg) or showing any other adverse event. Patients were not allowed to take any other antihypertensive drug during the study. The study was conducted in accordance with the ethical principles of the Helsinki Declaration.

## Statistical Analysis

The Statistical Package for the Social Sciences (SPSS) program version 19.0 was used to perform the statistical analyses. Descriptive statistics were used to show the frequencies and percentages. Systolic and diastolic blood pressure measurements and percentage of systolic and diastolic blood pressure reduction were shown as Mean±SD. Pared student t-test was performed to compare baseline blood pressure readings with the readings after intervention and also one-way ANOVA was performed to compare study parameters between each treatment groups. Chi-square was used to compare gender, history of smoking and high serum cholesterol between treatment groups. The analysis of covariance (ANCOVA) for repeated measures was performed to identify the interaction between study categories and systolic and diastolic blood pressure changes. Confidence interval was considered as 0.95 and p values lesser than 0.05 were considered as significant.

## Results

Among 478 hypertensive cases, the mean age was 50.27±5.17 years (Median age was 50 years and IQR was 9 years). Table 1 presents systolic and diastolic blood pressures and patients’ demographic data. There was no significant differences between the three treatment groups in regard to gender, history of diabetes mellitus (DM), smoking and elevated serum cholesterol levels. The Amlodipine/losartan group were significantly younger than the other two groups (P=0.03).

**Table 1:** Distribution of Age, Gender, History of Diabetes Mellitus, Smoking, Elevated Serum Cholesterol Levels, Systolic and Diastolic Blood Pressure in the Population as a Whole and Each Treatment Subgroup at Baseline.

	Amlodipine/HCTZ group(N=159)	Losartan/ HCTZ group (N=155)	Amlodipine/ Losartan group (N=164)	Total(N=489)	P value
**Age (years)**	50.0±5.31	50.82±4.90	49.99±5.27	50.28±5.17	0.03
**Systolic BP† (mmHg)**	169.90±4.07	169.80±4.10	169.94±4.07	169.88±4.07	0.99
**Diastolic BP† (mmHg)**	105.29±3.61	105.19±3.61	88.78±6.75	105.29±3.59	0.97

Among the study population, baseline systolic and diastolic blood pressures were slightly higher in men than women but this difference was not significant as shown by (P=0.79) for systolic blood pressure and (P=0.92) for diastolic blood pressure. No significant difference was observed between age of men and women in this study (P=0.18).

Independent t-test revealed no significant difference in age between diabetic and non-diabetic patients (P=0.93). Also no significant difference was found in mean systolic and diastolic blood pressures between diabetic and non diabetic cases; (P=0.81) for systolic blood pressure and (P=0.52) for diastolic blood pressure. Among the 99 diabetic patients there was no difference between the number of men and women (P=0.84). Diabetic patients used to smoke significantly more than the non-diabetics (38.4% of diabetics and 16.6% of non- diabetics were smokers) (P<0.01). Chi-square test was used to identify the relationship between diabetes and high serum cholesterol levels. High serum cholesterol levels were detected in 94(96%) of diabetics which was significantly higher than 6(1.6%) of non-diabetics (P<0.01). Metabolic syndrome was found in 92 (25.5%) of non-diabetics while none of the diabetics exhibited metabolic syndrome, which was found to be significantly higher (P<0.01). In respect of response to treatment there was no relationship among diabetics (P =0.57).

There was no relationship between response to treatment and gender, smoking, serum cholesterol level, metabolic syndrome and history of diabetes (Table 2).

**Table 2:** Comparison of Study Variables Among Different Categories

		Age	Systolic BP	Diastolic BP
**Gender**	Male	50.1±5.3	170.0±4.1	105.4±3.5
	Female	50.8±5.2	169.9±4.1	105.3±3.6
	P value	0.18	0.79	0.92
**Diabetes mellitus**	Yes	50.4±5.5	169.9±4.1	105.5±3.6
	No	50.5±5.2	169.9±4.1	105.3±3.6
	P value	0.90	0.88	0.59
**Smoking**	Yes	50.1±5.3	169.7±4.0	105.7±3.5
	No	50.6±5.3	170.0±4.1	105.23±3.61
	P value	0.44	0.49	0.22
**Elevated serum cholesterol levels**	Yes	50.5±5.4	169.7±4.13	105.5±3.6
	No	50.4±5.2	170.0±4.05	105.3±3.6
	P value	0.86	0.58	0.52
**Metabolic syndrome**	Yes	50.2±5.2	170.0±4.29	104.9±3.6
	No	50.5±5.3	169.9±4.01	105.4±3.6
	P value	0.57	0.80	0.24

There was a significant difference in systolic blood pressure reductions between the three treatment groups (P<0.001). There was also a significant difference between groups in reducing diastolic blood pressure (P<0.01). The highest systolic blood pressure reduction was found in treatment with Amlodipine/losartane followed by amlodipine/HCTZ and losartane alone (Table 3). The ANCOVA analysis revealed that only treatment regimen had a significant effect on blood pressure reduction (P=0.01).

**Table 3:** Comparison of Systolic and Diastolic Blood Pressure at Baseline and after Treatment

	Amlodipine /HCTZ (N†=159)		Amlodipine/Losartan (N†=164)		Losartan /HTCZ (N†=155)	
	Baseline	After	Baseline	After	Baseline	After
**Systolic BP**	169.9±4.1	137.7±5.6	169.9±4.1	135.0±5.4	169.8±4.1	140.6±8.5
**P value**	<0.001		<0.001		<0.001	
**Systolic BP reduction (%)**	32.17±8.58 (18.9%)		35.0±3.6 (20.5%)		29.7±9.4 (17.5%)	
**Diastolic BP**	105.3±3.6	88.9±6.2	88.8±6.8	84.67±4.4	105.2±3.6	89.1±6.8
**P value**	<0.001		<0.001		<0.001	
**Diastolic BP reduction (%)**	32.5±8.4 (30.9%)		29.6±6.2 (28.2%)		34.8±9.2 (33.3%)	

Repeated measure test revealed significant difference in reduction rate of both systolic and diastolic pressure (P<0.001). Repeated measure test also showed that there is a significant relation in interaction between the before and after blood pressures (P<0.001).

## Discussion

Combination therapy is proved to have better effects on blood pressure control in patients with stage 2 hypertension (9-12). It was previously shown that the effect of combination therapy would be five times greater than doubling the dose of a single antihypertensive drug (13). This study found that all combinations resulted in 38% reduction in systolic blood pressure. A recent study performed to compare the effect of Amlodipine/Losartan (5mg/100mg) combination therapy with single therapy with Amlodipine in Korea reported response rate of 90% for Amlodipine/Losartan treatment (14). Although a lower dose of Losartan was administered in this study, 89% response rate was acheived with Amlodipine/Losartan treatment. This finding indicates that in combination therapy with Amlodipine, the dose of Losartan can be reduced by 50% to achieve similar response rate when administered at 100mg bid. In this study, response to treatment in Amlodipine/HCTZ treatment group was 72% that was similar to the study by Oparil et al (1996) responce rate of 71% (15). Response rate to Losartan/HCTZ was 54% in this study. In a study by Bonner et al. (2009) response rate to combination therapy with 100mg Losartan plus 25mg HCTZ in stage 1 hypertensive patients was 53.8%.(16) In our study reducing Losartan dose by half, if combined with HCTZ, will not affect the response rate to combination therapy.

Amlodipine/losartane combination therapy resulted in the highest systolic blood pressure reduction (20.5%) in this study. In a study by Kim et al. (17) systolic BP was reduced by 22.1% after 8 week treatment with 50mg Amlodipine/Losartan treatment. Although in their study Losartan was administered at a higher dose (50mg compared with 25mg in our study). This difference can be due to the usage of higher losartane doses in the Kim et al.(17) study or a larger study population in this study (73 patients in Kim et al. study vs 164 patients in this study). In this study systolic blood pressure was reduced significantly with amlodipine/losartane combination therapy. Systolic BP reduction with Losartan/HCTZ combination therapy was reported 15% in the Bonner et al. (16) study, which was performed on a larger population (n=4143) with the mean BP being in the range of stage 1 hypertension.(16) Systolic BP was reduced by 9% in Opal et al. (15) study, where the mean systolic BP was 159.0 mmHg which was lower than 169.88 mmHg in this study.

In this study combination therapy with Losartan/HCTZ resulted in the highest (33.3%) reduction in diastolic BP. In the study by Bonner et al. (16), combination therapy with Losartan/HCTZ resulted in 12.9% reduction in diastolic BP which might be due to the study population, stage 1 hypertensive patients. (16) In this study combination therapy with Amlodipine/Losartan resulted in 28.2% reduction in diastolic BP. In the study by Kim et al. (17) diastolic BP was reduced by 15.5% after 8 weeks treatment with Amlodipine/Losartan. The findings of this study revealed that Amlodipine/Losartan could result in a more reduction in diastolic BP (17% vs 15.5% in the study by Kim et al.) (17). No recent study was found to compare the effect of ARB/HCTZ with CCB/HTCZ. The results of this study indicated that CCB/HCZ combination might not be a useful combination for reducing BP in stage 2 hypertension.

One of the limitations of this study was that there was no control on patients’ lifestyle including usual physical activity and food or drink intakes. It is recommended that further controlled trials be performed by selecting matched cases with similar lifestyle and BP.
